# Polyphenols and Organic Acids as Alternatives to Antimicrobials in Poultry Rearing: A Review

**DOI:** 10.3390/antibiotics10081010

**Published:** 2021-08-20

**Authors:** Federica Scicutella, Federica Mannelli, Matteo Daghio, Carlo Viti, Arianna Buccioni

**Affiliations:** Dipartimento di Scienze e Tecnologie Agrarie, Alimentari, Ambientali e Forestali, University of Florence, Piazzale delle Cascine 18, 50144 Florence, Italy; federica.mannelli@unifi.it (F.M.); matteo.daghio@unifi.it (M.D.); carlo.viti@unifi.it (C.V.); arianna.buccioni@unifi.it (A.B.)

**Keywords:** phenolic compounds, fatty acids, broiler, laying hens, antibacterial, anticoccidial, growth promoter

## Abstract

For decades antibiotics have been used in poultry rearing to support high levels of production. Nevertheless, several problems have arisen because of the misuse of antibiotics (i.e., antibiotic resistance, residues in animal products, environmental pollution). Thus, the European Union (EU) as well as the European Food Safety Authority (EFSA) promote action plans to diminish the use of antibiotics in animal production. Alternatives to antibiotics have been studied. Polyphenols (PPs) or organic acids (OAs) seem to be two accredited solutions. Phenolic compounds, such as phenols, flavonoids, and tannins exert their antimicrobial effect with specific mechanisms. In contrast, short chain fatty acids (SCFAs) and medium chain fatty acids (MCFAs), the OAs mainly used as antibiotics alternative, act on the pathogens depending on the pKa value. This review aims to collect the literature reporting the effects of these substances applied as antimicrobial molecules or growth promoter in poultry feeding (both for broilers and laying hens). Organic acids and PPs can be used individually or in blends, exploiting the properties of each component. Collected data highlighted that further research needs to focus on OAs in laying hens’ feeding and also determine the right combination in blends with PPs.

## 1. Introduction

The use of antibiotics in conventional farming has been the main strategy to protect animals from the insurgence of infections and to prevent epidemic diseases, increasing performance and promoting growth. This massive employment of drugs in animal production has led to the development of microorganism antibiotic resistance [1,2,3]. To overcome the selection of resistant bacteria, the European Union (EU) banned their use as growth promoter in 2006 [4], and since 2013 has developed an action plan to fight antibiotic resistance [5]. This plan advises the application of good animal management practices, monitoring the Member States’ drug employment, and supports the researcher community to find affordable alternatives to antibiotics

Alternatives to conventional antibiotics have been studied [6,7,8,9], and these substances are used in feeds as preventive or therapeutic measures [10,11]. Among them, literature reports that the use of plant secondary metabolites (i.e., polyphenols (PPs) and essential oils) and organic acids (OAs, i.e., short-chain fatty acids (SCFAs) and medium chain fatty acids (MCFAs)) may be valid solutions [7,12,13]. However, all medicated feeds will face more restrictive legislation, because in 2018 the EU released another group of regulations that will become functional in 2022. This new statement completely forbids the preventive use of antibiotics, except if appropriate alternatives are not available [10,11].

Polyphenols are naturally synthetized in different plant organs to protect against pathogens, thanks to their antibacterial and antifungal properties [14]. Hence, they could exert an antibiotic-like action in animal management [15,16]. For instance, plant-based herbal additives were studied as anticoccidials in poultry management [17]. Even though the observed effect was not comparable with that obtained with antibiotics, data highlighted positive effects on animal performance and intestinal lesion score for broilers fed with the natural products. Furthermore, an anti-inflammatory action was observed in the liver of broilers fed with a diet containing polyphenolic sources, such as milk thistle seeds, rich in silymarin [18]. Organic acids have been proven to have an antibiotic-like action and were recognized as safe for animal feeding by the EU [19]. They exert an antimicrobial activity on gut microorganisms [20] and improve protein digestibility and amino acids’ absorption [21].

Poultry is the most diffuse form of animal production in the world providing a protein source at a low cost, obtained in a very short production process; moreover, the small animal size allows for the management of a huge number of chickens close together. To keep supporting these high levels of production, antimicrobials are extremely important in poultry rearing [22]. Thus, they are usually employed to prevent the easy disease spread, considering the birds’ typical gregarious behavior and their habit of pecking at the ground. Consequently, this review recovered literature about PP-rich sources and OAs that exert an antibiotic-like action for poultry as antimicrobials.

## 2. Polyphenols

### 2.1. Chemical Characteristics

Plants synthesize secondary metabolites with antimicrobial properties to protect their organs from microbial infections or herbivorous grazing [23]. Generally, they are produced in special circumstances (i.e., after wounds or microbial infections) or in specific locations that demand more protection. Polyphenols constitute one of the most numerous and widely distributed groups of these natural products. More than 8000 phenolic compounds have been discovered and partially studied [24]. The classification of PPs was carried out over the course of several years, and they can be categorized in several ways [25]. The most accredited form of classification is to consider their chemical structure and properties [24]. All PPs feature from an aromatic ring plus one or more hydroxyl groups [26].

**Figure 1:**
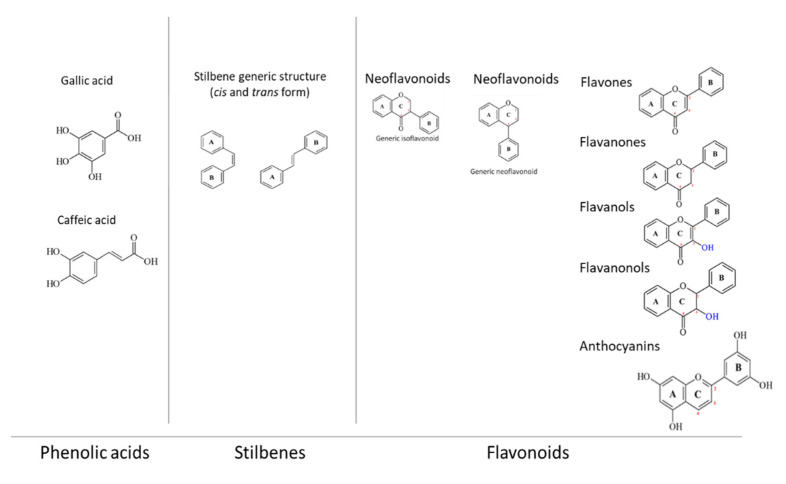
Examples of polyphenols applied as antimicrobials.

Three main groups can be identified: phenolic acids, flavonoids, and stilbenes (Figure 1). In addition, there are other PPs out of this classification that have a simple structure and are water-soluble. Phenolic acids are formed by a simple benzene ring (i.e., gallic acid or caffeic acid) and are rarely found in a free form but, usually, they are bound in plant cell walls or lignin [27,28]. The flavonoid group involves the greater number of PPs, nearly 5000 different molecules; all flavonoids are characterized by two aromatic rings linked by a 3-carbon bridge [29]. Moreover, they can be further classified into several subgroups: neoflavonoids, isoflavonoids, flavones, flavanols, flavanones, and anthocyanidins [30]. Stilbenes are constituted by two aromatic rings without a carbon bridge [31].

Tannins belong to the polyphenol class. They are distinguished into hydrolysable tannins (HT) and condensed tannins (CT). The first ones phenolic acids esterified to hydroxyl-groups, while the latter are flavan-3-ol polymers, each with different chemical activities [30].

### 2.2. Antimicrobial Activity

**Figure 2:**
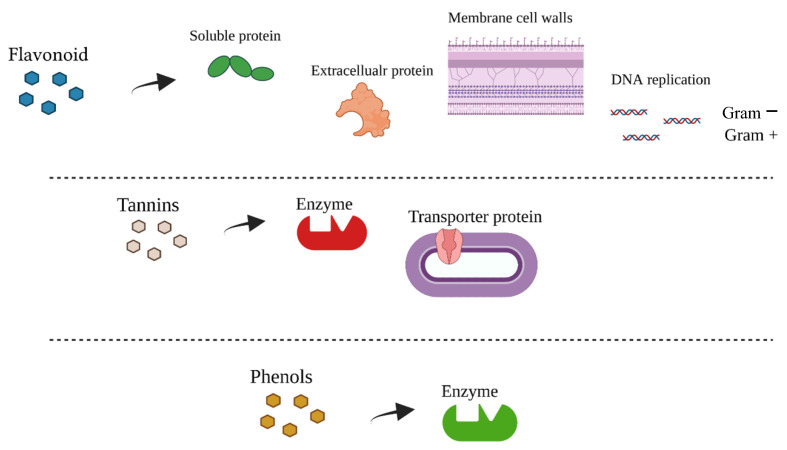
Flavonoid, tannin and phenol antimicrobial mechanisms of action.

In general, phenols exert their toxicity inhibiting enzymes because their oxidized compounds interact with sulfhydryl groups or with proteins, without a specific mechanism. Flavonoids can bind extracellular and soluble proteins (i.e., hydro-lases, oxidoreductases, DNA synthetases, RNA polymerases, phosphatases, protein phosphokinases, oxygenase, and amino acid oxidases [32]) or bacterial cell-walls, and the lipophilic one can destroy membrane [33]. In addition, they can inhibit DNA replication, either in Gram+ or Gram− bacteria [34]. Tannins act as antimicrobials since they deactivate microbial adhesins, enzymes, cell envelope transport proteins, and complexing with polysaccharides [33]. Particularly, CT can penetrate bacterial protein efficiently because of its lower molecular weight than HT [35] (Figure 2).

### 2.3. Poultry Feeding Application

Polyphenols have been already used in broilers and laying hens’ feeding with similar purposes: to improve bird health and performance, which are related [36,37]. However, since they are synthesized by plants also against grazing predation [13], they may have an anti-nutritional effect, limiting nutrient exploitation. From a chemical standpoint, PPs decrease feed intake and digestibility by binding dietary proteins and digestive enzymes [38]. To overcome this problem and to exploit the positive effects at the same time, the kind of PPs, the right level of inclusion, the eventual processing and synergic interaction with other additives are fundamental to know [7]. In fact, each kind of polyphenol can exert its effects in different ways (Table 1). For instance, HT and CT act differently against *Clostridium perfringens*. Literature reports that HT extract has a bactericidal activity while CT extract shows a bacteriostatic effect instead [39].

A common issue in using antibiotics in laying hens’ management is the transfer of chemical residues into the eggs. Nevertheless, in the USA they are still used to improve egg quality and quantity, particularly at the end of the deposition curve when production is less efficient. Thus, polyphenol sources seem to be a well-fitting solution because they have an antibiotic-like action and are residual-free [49]. For instance, strawberry guava leaf extract (a flavonoid source) used as feed additive can exert a double effect: an antimicrobial activity on the animal and an antioxidant activity on the eggs’ shelf-life [40]. *Escherichia coli* infection is a critical aspect of poultry rearing, because it decreases the production and increases the animal mortality [50]. Flavonoids and tannins, contained in crude extract of sambiloto (*Andrographis paniculate*) leaves, improve performance, even during the infection [41]. In fact, the challenged birds fed the integrated diet reduced their food consumption compared with the control, because the infection progression was inhibited, and feed digestion was more efficient. In addition, the andrographolide flavonoid from sambiloto increased the egg production due to its antibacterial effect. The diet inclusion of sambiloto crude extract as a polyphenol source led to lower food consumption with higher performance, ameliorating the feed conversion [41].

Magnolol is the main polyphenol extract from *Magnolia* L. root and stem bark, which exerts anti-inflammatory, antibacterial, anti-tumoral, and antioxidant activities [29,51,52,53]. In laying hens, a diet with a 200 mg/kg concentration of magnolol extract, administered in the late period, can improve hepatic lipid metabolism and intestinal mucosa barrier function, acting on the enteric tissue morphology (diet inclusion levels of 100 mg/kg, 200 mg/kg, and 300 mg/kg) [42]. In addition, quality of fresh and stored eggs and laying performance were ameliorated. Additionally, dandelion and marigold flowers, dried calendula, and basil leaves in hens were tested as sources of PPs, with two different levels of inclusion in the diet (10 g/kg and 30 g/kg) [43]. According to the literature, all these matrices reported positive responses as antimicrobial and anti-inflammatory agents due to their flavonoid content, and the effect against *Escherichia coli* counts was successfully exerted with the highest concentrations (30 g/kg).

Oregano essential oil, extracted from *Lippia origonoides*, was tested as an antimicrobial in laying hens because it is rich in thymol (8%) and carvacrol (4.9%), which act on the pathogen cell wall proteins [49]. The following conditions were tested: control without bacteriocin or PPs; 50 ppm zinc bacteriocin additive; 80 ppm oregano essential oil; and 150 ppm oregano essential oil. The feed treatment with 150 ppm oregano essential oil, without chemical additions, had the best bird feed intake and egg production, with the lowest mortality. Moreover, this treatment did not change egg nutritional quality [44].

Based on positive results from using *Petiveria alliacea* to improve bird growth and performances, the extract of its leaf and root were tested in growing pullets [45]. The anticoccidial and antimicrobial effects were exerted by different bioactive compounds (saponins, alkaloids, sulphur compounds, PPs) and the efficiency was higher with root extract than leaf extract. In fact, these matrices can act on *Eimeria* replication in feces, and on the total bacteria count in gut. Specifically, as anticoccidials, tannins, flavonoids, and phenols mitigated the parasitic infectious intensity thanks to their antioxidant activity. Instead, as natural antimicrobials they destroy microbial cell structure [45].

The advantage in using PPs as additives is related not only to animal health, but also to human health, reducing meat contamination. Human campylobacteriosis is associated with contaminated chicken meat consumption in the 50–80% of cases [54]. In fact, *Campylobacter*, which is present in chicken gut, could infect meat during slaughtering and carcass processing. Several authors tested polyphenol-rich matrices, such as polyphenolic extract from spray-dried olive mill wastewater [55], chestnut inner shell extract [56], or grape seed extract [57], as antimicrobials on the meat to lower the microbial count of *Campylobacter*. Thus, the effect of olive mill wastewater polyphenol extract and dehydrated olive cake on *Campylobacter* was evaluated in growing broilers (49 days of trial) [46]. Fecal samples were collected at different times during the experimental period. However, only after the 28th day of administration was the antimicrobial effect observed with both treatments. The olive mill wastewater extract, which had the higher polyphenol content, was more efficient in comparison to the olive cake. Moreover, unlike other olive by-products, both olive mill wastewater extract and dehydrated olive cake significantly improved animal performance [46].

Among the PPs extracted from vegetal matrices, one of the most common is curcumin, which exerts antioxidant, anti-inflammatory, antimicrobial, and gastroprotective activities [58] and a coccidiostat effect [59]. Using a combination of curcumin and a commercial microencapsulated phytogenic product (based on thymol, cinnamaldehyde and carvacrol), the broiler performance was increased through improving the positive intestinal flora. Hence, gut mucosa was protected from coccidia and bacteria (e.g., *Eimeria* and *Escherichia* spp.) proliferation. In addition, an increase in polyunsaturated fatty acids (FAs) with a lipid peroxidation decrease in meat was observed, improving meat quality [47]. Similarly, curcumin with yucca (*Yucca schidigera*) extract, a resveratrol and yuccaloid source, were tested with the intention of enhancing the antimicrobial effect [48]. Similar to the previous case, curcumin exerted an antimicrobial effect, inhibiting bacterial reproduction and exerting an anti-inflammatory action, improving animal performance. The yucca anticoccidial effect was exerted by the presence of saponins, due to their ability to bind with pathogen membrane cholesterol. Furthermore, quality and shelf-life of meat were improved. Particularly, curcumin was principally involved in producing a high content of polyunsaturated FAs, because it improved the efficiency of desaturase enzymes and reduced lipid peroxidation, thanks to its antioxidant properties. In addition, yuccaloids decreased saturated FA concentration, affecting the lipogenic enzyme Δ^9^-desaturase activity that converts saturated FAs into monounsaturated FAs, such as low-density lipoprotein (LDL) that is involved in cardiovascular disease risk [48].

## 3. Organic Acids

### 3.1. Chemical Characteristics

**Figure 3:**
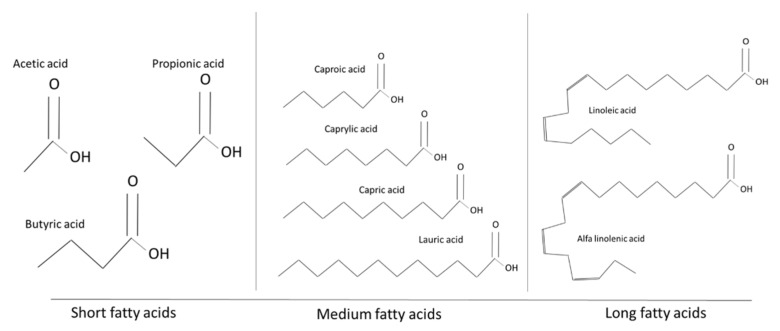
Examples of organic acids applied as antimicrobials.

Organic acids are organic carboxylic acids with a general structure R-COOH. They are classified on the basis of chain length (Figure 3). Particularly, for monogastrics, there are several OAs defined as “essential” because they cannot be synthetized by the gut microbiota. Short-chain fatty acids (C1–C7, i.e., formic, acetic, propionic, and butyric acids) are produced in the gut tract during feed fermentation [60], whilst MCFAs (C8–C12, i.e., caproic, caprylic, capric, and lauric acids) and long chain FAs (LCFA; C13–C32, i.e., linolenic and linoleic acids) need to be taken with the diet. In nature, most OAs are esterified with glycerol to form triglycerides and phosphoglycerides or their derivates. One molecule of glycerol can bind up to three molecules of FA, one for each hydroxyl attach site (position SN1, SN2 and SN3 on the glycerol carbon chain), forming a triacylglycerol. The SN1 and SN3 positions are chemically equivalent. Once ingested, triglycerides undergo lipolysis in the intestine, leading to the formation of diglycerides, monoglycerides, free FAs and free glycerol.

### 3.2. Antimicrobial Action

**Figure 4:**
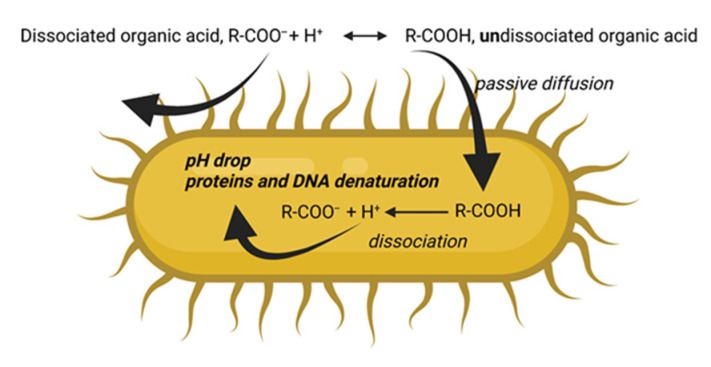
Example of organic acids’ antimicrobial mechanism of action.

Short-chain fatty acids (i.e., propionic (C3) and butyric (C4)) are principally related to antimicrobial activity, as well as to productive performance improvement [21,61]. In particular, the antimicrobial effect is associated with an acid dissociation constant (pKa) between 3 and 5. When OAs reach the gut, they alter the environment by decreasing pH. This acidification inhibits bacteria in nutrients recovery, which are preserved for the host. Moreover, a lower pH breaks down cholesterol and levels up calcium, phosphorus, and magnesium in serum due to an enhanced absorption [62]. Even MCFAs (C8–C12) are involved in antimicrobial activity, but their mechanism of action is different. Their pKa is about 4.9 [63] and those with a lower molecular weight are more efficient. They can easily penetrate semipermeable peptidoglycan (Gram+ bacteria) or phospholipid (Gram− bacteria) membrane in the undissociated form, then the dissociation in proton and anion carries out into the cytoplasm, lowering pH to induce the cell to collapse [64,65] (Figure 4).

### 3.3. Poultry Feeding Application

In poultry feeding, OA supplementation has been studied with positive effects (Table 2). However, the OA administration form is a common problem that the feed industry needs to solve to improve OA nutritional availability. The use of OA salts (with sodium, potassium, or calcium) is the first solution, because it provides OAs in a solid form with a better smell [20,66]. Microencapsulation is another solution [67,68], because the undegradable film that envelopes the OA particles allows a more effective release of these substances. Thus, the beneficial action is promoted along the entire gut tract. Organic acid efficiency is affected by molecular weight, pKa value, form (undissociated or dissociated) and the specific antimicrobial activity (targeted microorganism) [65,69].

For decades, OAs have been used in poultry production to improve meat quality and conservation [71,74]. In recent years, the addition of these substances in feeding has also been increasingly applied as an alternative to antibiotics. An example is glycerol-monolaurate that has been successfully used in poultry feeding and on meat to improve the nutritional quality. Thus, a 300 mg/kg content of glycerol-monolaurate can exert a growth-promotion action similar to antibiotics (levels of inclusion of 100 mg/kg, 200 mg/kg, and 300 mg/kg) [70]. In fact, this monoglyceride affected *Escherichia coli* abundance and *Eimeria* spp. oocyst abundance and proliferation.

A combination of OAs can also be used. For instance, a blend of SCFAs and MCFAs and a low dose of ß1-4 mannobiose against *Salmonella enterica* serovar Typhimurium was evaluated in broilers [69]. However, the dose of 3 g/kg improved the performances only minimally, with a stable activity of the probiotic flora. The authors concluded that the concentration of OAs was too low to show an effect on the microbiota [69]. Elsewhere, an evident antibacterial effect was observed already in the first days of the experimental period using a blend of OAs with a 0.2% level of inclusion [71]. Particularly, SCFAs decreased the abundance of *Enterococcus* spp., which typically infect one-day-old birds. Likewise, these were more efficient in reducing *Faecalibacterium* in adults, compared with MCFAs. In addition, *Lactobacillus* and *Bifidobacterium* genera were found to be more abundant, improving immunity-response and, subsequently, meat quality. Nevertheless, *Lactobacillus crispatus* and *Lactobacillus salivarius* were the most abundant species in both treatment groups, exerting different beneficial effects (antibacterial, growth promoter and healthy) [71].

Among the most recent strategies to enrich diets with OAs, the literature reported how the dietary fermentable fiber fraction can be exploited to produce bioactive FAs in the animal gut, exerting an antimicrobial effect [75]. Wheat bran, a by-product of wheat milling, was added to feed (1%) to evaluate the effect against *Salmonella* [72]. Previously, the particle-size antimicrobial effect was tested in vitro. Then, a wheat bran with a 280 µm particle size was evaluated in vivo on broilers. The chosen wheat bran granulometry led to a fast fermentation that produced butyric acid. This OA is involved in the downregulation of the genes of the *Salmonella* pathogenicity island I (SPI-1) [76] that eases *Salmonella* propagation in the animal gut, exerting an antimicrobial effect. In addition, this strategy improved animal performance, in accordance with other similar studies [77,78]. Elsewhere, the antimicrobial action exerted by the LCFAs produced from cranberry pomace fermentation highlighted a double positive effect [73]. The high α-linolenic acid (21.0%) and linoleic acid (39.7%) pomace concentrations hindered encephalomalacia onset and promoted a better immunologic response against infectious bursal disease virus (IBDV) and Newcastle disease virus (NDV). Moreover, its fiber content favored *Ruminococcus caecal* bacterium presence, involved in the plant cell wall breakdown. Lastly, this kind of FA levels up the meat’s nutritional quality [73].

## 4. Blends of Polyphenols and Organic Acids

The use of different kinds of substances added to animal feed as a blend could exert interesting effects, enhancing the activity of each component (Table 3).

Chestnut tannin blended with SN1 monoglycerides affects the gut with an antimicrobial action, providing energy to enterocytes, increasing villi growth and then, mitigating the negative effect of tannins [80,81,82]. Similarly, diet palatability, weight gain or feed efficiency were not compromised, despite the tannin’s astringent action [67]. Instead, a blend of glycerol monolaurate (monoglyceride of lauric acid), curcumin and cinnamaldehyde (polyphenol compounds) was evaluated as feed additive for broiler diet, substituting conventional growth promoters [68]. Specially during the starter diet period, the authors found that the tested levels of inclusion (276 mg of curcuminoids/kg, 156 mg cinnamaldehyde/kg, 297 mg glycerol-monolaurate/kg) exerted a toxic effect on the animal, resulting in a growth reduction. In contrast, despite its low efficiency as growth promoter, the blend seemed to be optimal as an antimicrobial or anticoccidial additive, since curcumin decreased bacterial reproduction, cinnamaldehyde undermined *Eimeria* oocyst viability, and glycerol monolaurate interfered with pathogen activity. A similar situation was observed when studying a blend of cinnamaldehyde and thymol (from essential oils) with or without sodium butyrate at different levels of inclusion (only essential oil addition of 50 mg/kg and 100 mg/kg; or their combination with 1 g/kg of sodium-butyrate) [79]. Compared with the control, performances did not improve in any treated group. However, other beneficial effects were detected within the tested conditions. The most efficient treatments were the ones with a low phenolic compound content (50 mg essential oil blend/kg) and 1 g/kg of OA inclusion. In fact, *Salmonella* contamination was lowered in both caeca and fecal samples. Probably, other doses need to be tested to observe an antimicrobial effect together with growth promotion. In addition, the carcass was not contaminated with pathogens, reducing the chance of infectious transmission to humans, according to the original employment of OAs as a food additive for preservation [71,74]. In the same way, to avoid meat contamination during slaughtering, several plant-based or OA-based commercial feed additives were tested in vivo to evaluate their efficiency during the livestock period until slaughtering [54]. However, no antimicrobial activity was confirmed until the period immediately before slaughtering. The authors concluded that the OAs or the other bioactive compounds were probably previously absorbed or metabolized in a gut tract different to the caeca [54].

A blend of OAs (citric, fumaric, sorbic, and malic acids) with tannins, curcumin, and essential oils at different levels of feed inclusion (250 mg/kg, 500 mg/kg, and 1000 mg/kg) was tested [36]. In each treated group, performances were not comparable with the control including antibiotics. On the 22nd day of life animals were challenged with aflatoxin, simulating a feed contamination. In litter feces, lower counts of heterophiles, lymphocytes, and monocytes, and a lower bacteria cell count were observed, and in breast meat a lower lipid peroxidation was found. Despite displaying no higher growth performances, both intestine health and meat shelf-life were improved by the blend of OA and vegetal bioactive molecules, confirming the antimicrobial effect of SCFAs and the antioxidant activity of phenolic compounds. In contrast with the previous papers reported, a blend of SCFAs and MCFAs with thymol, cinnamaldehyde and essential oil of eucalyptus can exert a synergic effect as antimicrobials and as growth promoters [83].

## 5. Conclusions

Either PPs or OAs can be successfully applied in poultry feeding as a preventive alternative to antibiotics, and as growth promoters. In accordance with the newest EU regulation [10,11], more research is needed to exploit these alternatives to antibiotics together with a therapeutic purpose. Generally, PPs’ and OAs’ antimicrobial action is exerted on the intestinal microbiota. These lead to an energy saving that the host metabolism can employ in muscle production instead of in an anti-inflammatory reaction. Moreover, a gut health improvement evidences better and higher nutrient absorption.

Other uses of these substances in blends show different effects, depending on the molecule kind, the level of inclusion, and the interaction between PPs and OAs. In fact, blends exert an antimicrobial effect, but auxin action is not always observed. Thus, further investigations about the mechanism of action could be interesting to understand how to properly exploit each product for a specific purpose or in different areas of livestock management.

From our knowledge, even though PPs are also used in laying hen rearing, there is a lack of investigation about the employment of OAs in this sector. In addition, an interesting application, not explored in this paper, would be the employment of PPs and OAs as food preservatives.

The use of PPs or OAs in poultry feeding is beneficial not only as an alternative to antibiotics for animal welfare and environment preservation but also for the product (e.g., egg or meat) quality, improving food nutritional profile, safety, and shelf-life.

## Figures and Tables

**Table 1:** Example of polyphenols studied as antimicrobials.

Poliphenols	Quantity	Effects	References
Hydrolizable tannins	n.a	Bactericidial against *Clostridium perfringens*	Redondo et al., 2015 [39]
Condensed tannins	n.a.	Bacteriostatic against *Clostridium perfringens*	Redondo et al., 2015 [39]
Flavonoids	<0.2 g/kg	Antimicrobial against *Salmonella* spp. and *Escherichia coli*	Dos Santos et al., 2020 [40]
Andrographolide flavonoid and tannins	0.3%	Antibacterial against *Escherichia coli*	Hidanah et al., 2020 [41]
Magnolol	200 mg/kg	Antimicrobial against *Escherichia coli*	Chen et al., 2020 [42]
Flavonoids	30 g/kg	Antimicrobial against *Escherichia coli*	Balenović et al., 2018 [43]
Thymol and carvacrol	8% and 4.9%	Antimicrobial	Ramirez et al., 2021 [44]
Tannins, flavonoids and phenols	0.45 g/L	Anticoccidial against *Eimeria* oocyst antimicrobial	Oyeleke et al., 2021 [45]
Polyphenols	0.263 g/kg; 0.556 g/kg	Antimicrobial against *Campylobacter* spp.	Branciari et al., 2016 [46]
Curcumin, thymol, cinnamaldehyde and carvacrol	50 mg/kg and 100 mg/kg	Anticoccidial against *Eimeria* oocyst and antibacterial against *Escherichia coli*	Galli et al., 2020a [47]
Curcumin, resveratrol, yuccaloids	100 mg/kg and 250 mg/kg	Antimicrobial and anticoccidial against *Eimeria*	Galli et al., 2020b [48]
Curcumin, thymol, cinnamaldehyde and carvacrol	50 mg/kg and 100 mg/kg	Anticoccidial against *Eimeria* oocyst and antibacterial against *Escherichia coli*	Galli et al., 2020a [47]

**Table 2:** Examples of organic acids studied as antimicrobials.

Organic Acid	Quantity	Effects	References
Glycerol-monolaurate	300 mg/kg	Antimicrobial against *Escherichia coli* and anticoccidial against *Eimeria* oocyst	Fortuoso et al., 2019 [70]
Short and medium fatty acids	3 g/kg	Antimicrobial against *Salmonella enterica*	Aljumaah et al., 2020 [69]
Short and medium fatty acids	0.20%	Antibacterial against *Enteroccocus*	Dauksiene et al., 2021 [71]
Fatty acids produced by wheat bran fermentation	1% with 280 µm particle size	Antimicrobial against *Salmonella*	Vermeulen et al., 2017 [72]
Long-chain fatty acids by cranberry pomace fermentation	αlinolenic acid 21% and linoleic acid 39.7%	Improvement of immunologic response against infectious bursal disease virus (IBDV) and Newcastle disease virus (NDV)	Islam et al., 2020 [73]

**Table 3:** Examples of blends of polyphenols and organic acids studied as antimicrobials.

Blends of Polyphenols and Organic Acids	Quantity	Effects	References
Chestnut tannin extract and SN1 monoglycerides (a mix of organic acids from C4:0 to C12:0)	2 g/kg and 1 g/kg; 1 g/kg and 2 g/kg	Antimicrobial against *Clostridium perfringens*, *Salmonella typhymurium*, *Escherichia coli* and *Campylobacter jejuni*	Mannelli et al., 2019 [67]
Glycerol-monolaurate with curcumin and cinnamaldehyde	297 mg/kg, 276 mg/kg, and 156 mg/kg	Against *Eimeria* oocysts viability	Galli et al., 2021 [68]
Thymol and sodium butyrate	50 mg/kg and 1 g/kg	Against *Salmonella* counts	Cerisuelo et al., 2014 [79]
Commercial blend	n.a.	Antimicrobial against *Campylobacter*	Guyard-Nicodème et al., 2016 [54]
Citric, fumaric, sorbic, and malic acids	250 mg/kg, 500 mg/kg, and 1000 mg/kg	Antimicrobial *Eimeria* and *Escherichia coli*	Armanini et al., 2021 [36]

## Abbreviations

CT: condensed tannin; FA: fatty acids; HT: hydrolysable tannin; LCFA: long chain fatty acid; LDL: low density lipoprotein; MCFA: medium chain fatty acid; OA: organic acid; PP: polyphenols; SCFA: short-chain fatty acid.
